# Inspiratory muscle training improves exercise capacity with thoracic load carriage

**DOI:** 10.14814/phy2.13558

**Published:** 2018-02-09

**Authors:** Ren‐Jay Shei, Robert F. Chapman, Allison H. Gruber, Timothy D. Mickleborough

**Affiliations:** ^1^ Division of Pulmonary, Allergy, and Critical Care Medicine Department of Medicine University of Alabama at Birmingham Birmingham Alabama; ^2^ Department of Kinesiology School of Public Health‐Bloomington Indiana University Bloomington Indiana

**Keywords:** Diaphragm fatigue, flow limitation, performance, respiratory muscles, ventilation

## Abstract

Thoracic load carriage (LC) exercise impairs exercise performance compared to unloaded exercise, partially due to impaired respiratory mechanics. We investigated the effects of LC on exercise and diaphragmatic fatigue in a constant‐load exercise task; and whether inspiratory muscle training (IMT) improved exercise capacity and diaphragmatic fatigue with LC. Twelve recreationally active males completed three separate running trials to exhaustion (*T*_lim_) at a fixed speed eliciting 70% of their $\dot{V}$O_2max_. The first two trials were completed either unloaded (UL) or while carrying a 10 kg backpack (LC). Subjects then completed 6 weeks of either true IMT or placebo‐IMT. Posttraining, subjects completed an additional LC trial identical to the pretraining LC trial. Exercise metabolic and ventilatory measures were recorded. Diaphragm fatigue was assessed as the difference between preexercise and postexercise twitch diaphragmatic pressure (*P*_di, tw_), assessed by bilateral stimulation of the phrenic nerve with esophageal balloon‐tipped catheters measuring intrathoracic pressures. *T*_lim_ was significantly shorter (*P *<* *0.001) with LC compared with UL by 42.9 (29.1)% (1626.5 (866.7) sec and 2311.6 (1246.5) sec, respectively). The change in *P*_di, tw_ from pre‐ to postexercise was significantly greater (*P *=* *0.001) in LC (−13.9 (5.3)%) compared with UL (3.8 (6.5)%). Six weeks of IMT significantly improved *T*_lim_ compared to pretraining (*P = *0.029, %Δ +29.3 (15.7)% IMT, −8.8 (27.2)% Placebo), but did not alter the magnitude of diaphragmatic fatigue following a run to exhaustion (*P *>* *0.05). Minute ventilation and breathing mechanics were unchanged post‐IMT (*P *>* *0.05). Six weeks of flow‐resistive IMT improved exercise capacity, but did not mitigate diaphragmatic fatigue following submaximal, constant‐load running to volitional exhaustion with LC.

## Introduction

Carrying a load on the thoracic cavity (load carriage, LC) using apparatuses such as backpacks and load‐bearing vests is often required to transport equipment and supplies in a variety of occupational and recreational settings (Muza et al. 1989; Brown and McConnell 2012; Dominelli et al. 2012). Thoracic LC impairs exercise performance compared with unloaded exercise by imposing extra stress on the cardiopulmonary and limb locomotor muscle systems (Muza et al. 1989; Brown and McConnell 2012; Dominelli et al. 2012). For example, thoracic LC reduces forced vital capacity (FVC) and forced expiratory volume in 1 sec (FEV_1.0_), and increases the elastic power output of breathing (Muza et al. 1989; Bygrave et al. 2004; Legg and Cruz 2004; Dominelli et al. 2012). Consequently, minute ventilation ($\dot{V}$_E_), heart rate, and oxygen consumption ($\dot{V}$O_2_) are elevated during slow‐ and moderate‐paced walking with thoracic LC compared to unloaded exercise (Majumdar et al. 1997; Ricciardi et al. 2008; Phillips et al. 2016). More recently, thoracic LC was shown to increase both $\dot{V}$_E_ and breathing frequency (*f*_b_) during exercise, with a concomitant reduction in tidal volume (*V*_T_) and end‐inspiratory lung volume compared to unloaded exercise at a matched $\dot{V}$O_2_ (Phillips et al. 2016). Ultimately, these effects on pulmonary and respiratory muscle function may alter ventilatory mechanics and accelerate respiratory muscle fatigue (Muza et al. 1989; Brown and McConnell 2012; Dominelli et al. 2012; Faghy and Brown 2014, 2016; Faghy et al. 2016). While thoracic LC has been shown to induce global respiratory muscle fatigue, measured by postexercise reductions in maximal voluntary mouth pressure, presently no data are available on the effects of thoracic LC exercise on specific diaphragmatic fatigue and its impact on constant‐work rate exercise to exhaustion.

Studies conducted by Faghy and Brown (2014, 2016), Faghy et al. (2016), and Phillips et al. (2016), clearly indicate that thoracic LC places extra stress upon the respiratory muscles. It is therefore reasonable to propose that training the respiratory muscles may be a strategy to ameliorate the detrimental effects of thoracic LC upon human performance. Flow‐resistive inspiratory muscle training (IMT) has been shown to improve respiratory muscle function and exercise performance, and to lower the oxygen cost, heart rate, blood lactate concentration, and the perceptual response during constant load exercise in recreational runners (Mickleborough et al. 2010). To date, only one study has investigated the effects of IMT on exercise performance with thoracic LC (Faghy and Brown 2016). While their findings that IMT improved performance in a 2.4 km running time‐trial and reduced the amount of global inspiratory muscle fatigue following exercise were novel, their primary measure of inspiratory muscle fatigue was volitional maximal inspiratory mouth pressure (*P*_Imax_). However, the most objective method of evaluating contractile fatigue of the diaphragm, the primary muscle of inspiration, is the measurement of transdiaphragmatic pressure in response to bilateral phrenic nerve stimulation (*P*_di, tw_), since it eliminates the influence of motivation (ATS/ERS Statement on Respiratory Muscle Testing, 2002; Janssens et al. 2013). To our knowledge, there are no published data characterizing the impact of IMT on postexercise diaphragmatic fatigue, as assessed by non‐volitional *P*_di, tw_.

Similarly, there are no data describing whether flow‐resistive IMT affects exercise capacity with thoracic LC as assessed by running at a fixed speed to the point of volitional exhaustion, and whether the contribution of diaphragmatic fatigue at task failure plays an important role. Since thoracic LC exercise in many occupational and recreational settings not only requires the ability to perform exercise quickly, but to sustain exercise over a long period of time, investigating strategies to optimize both exercise performance and exercise capacity with thoracic LC are warranted.

Therefore, the purpose of this study was to determine whether 6 weeks of flow‐resistive IMT would attenuate thoracic LC exercise‐induced diaphragmatic fatigue, and thereby improve exercise tolerance. Based on the findings of Faghy and Brown (2016), and Mickleborough et al. (2010), we hypothesized, (1) that the severity of diaphragmatic fatigue would be greater following thoracic LC exercise compared to unloaded exercise, and (2) that following flow‐resistive IMT the severity of diaphragmatic fatigue would be less, and thoracic LC running time to exhaustion would be enhanced.

## Methods

### Subjects

Twelve non‐smoking, recreationally active men with no prior history of pulmonary or cardiovascular disease (assessed by questionnaire) participated in this study (Table 1). Subjects met the criteria for classification as low risk for exercise testing by the American College of Sports Medicine risk stratification criteria (Pescatello et al. 2014). Participants exercised a minimum of 30 min, three times a week, and regularly participated in walking, running, hiking, jogging, or similar exercise. All subjects were instructed to continue their normal diet and regular exercise regimen throughout the duration of the study, to refrain from strenuous exercise for 24 h prior to each testing session, and to avoid caffeine and alcohol in the 8 h prior to each testing session. All testing procedures and the informed consent document were approved by the Institutional Review Board Human Subjects Committee. Written informed consent was obtained prior to subjects being enrolled in the study.

**Table 1:** Subject characteristics (*n *=* *12)

Variable	Pooled *n *=* *12	Inspiratory Muscle Training (IMT) *n *=* *6	Placebo (PLA) *n *=* *6
Age (years)	23.5 (5.3)	23.3 (3.6)	23.7 (7.0)
(Range)	(18–37)	(19–28)	(18–37)
Body mass (kg)	72.7 (6.0)	73.4 (3.5)	72.0 (8.1)
Height (cm)	179.7 (6.7)	181.5 (7.8)	177.9 (5.6)
Body mass index (kg·m^2^)	22.5 (1.8)	22.3 (1.3)	22.7 (2.3)
$\dot{V}$O_2max_ (mL·kg^−1^·min^−1^)	61.9 (6.0)	62.0 (7.9)	61.8 (4.0)
$\dot{V}$O_2max_ (L·min^−1^)	4.5 (0.5)	4.5 (0.5)	4.4 (0.6)
Weekly activity (hours)	9.2 (4.3)	8.7 (1.8)	9.7 (6.2)
Training compliance (%)	–	92.6 (8.4)	87.0 (9.7)

$\dot{V}$O_2max_, maximal oxygen consumption. Data are given as mean(SD).

### Study Design

Subjects completed four laboratory visits at the same time of day on separate occasions, separated by a minimum of 2 days. A repeated measured, randomized, double‐blind study design was implemented. Subjects were familiarized with all testing procedures prior to the experimental visits.

On the first laboratory visit (*visit 1*), anthropometric, exercise, and pulmonary function measures were collected. Maximal oxygen uptake ($\dot{V}$O_2max_) was determined using a graded exercise test on a motorized treadmill and was used to determine the running speeds that elicited an absolute $\dot{V}$O_2_ equal to 70% of $\dot{V}$O_2max_ while wearing a 10 kg weighted backpack (loaded, LC) and no backpack (unloaded, UL). An exercise intensity of 70% of $\dot{V}$O_2max_ was selected because this intensity is expected to be near, but below the threshold (>85% $\dot{V}$O_2max_) at which diaphragmatic fatigue has been documented to occur (Harms et al. 1997; Romer et al. 2006). Thus, we expected diaphragmatic fatigue to be absent in the unloaded condition based on previous findings (Harms et al. 1997; Romer et al. 2006), but intensity selected was expected to be high enough that the additional stress of LC may induce diaphragmatic fatigue. Subjects were then familiarized with the testing procedure by completing a familiarization trial. This trial consisted of a constant‐load run the point of volitional exhaustion with the weighted backpack (loaded) at the speed determined to elicit 70% of $\dot{V}$O_2max_. A fixed‐workload run to exhaustion was selected over other tests such as fixed‐distance time trials (in which exercise intensity is self‐selected by subjects) in order to allow for better experimental control to determine the effects of LC on diaphragmatic fatigue at the selected sub‐maximal workload, which was not expected to induce diaphragmatic fatigue in normal, unloaded exercise. We further note that tests such as fixed‐distance time trials or fixed‐time tests may not reflect the recreational and occupational demands that some people who undertake load carriage exercise must face. Subjects were blinded to all feedback during the exercise bout and instructed to run at the prescribed speed for the longest time possible until task failure.

Subjects then performed runs to volitional exhaustion at a speed equivalent to 70% of their $\dot{V}$O_2max_ during three separate occasions (*visits 2, 3, and 4*). *Visits 2 and 3* were completed in either the UL or LC condition in a randomized and counterbalanced order. Running time to exhaustion (*T*_lim_), exercise metabolic, and perceptual measures were recorded, and diaphragmatic strength was assessed pre‐ and postexercise using bilateral phrenic nerve stimulation to characterize the presence or absence of diaphragmatic fatigue. *Visit 4* was completed in a similar manner to *visits 2 and 3,* in a loaded condition 6 weeks after *visit 3,* following a period of either true IMT (IMT group) or placebo IMT (PLA group). Group assignment was randomized by non‐essential study personnel, and groups were matched for baseline pulmonary and respiratory muscle function and baseline running *T*_lim_.

### Graded Exercise Protocol (Treadmill)

Maximal oxygen uptake was assessed using a previously described protocol (Duke et al. 2014). Briefly, subjects began the test by running on a flat treadmill (0% grade) for 2 min, after which the treadmill grade was increased to 4% for 2 min, and incrementally by 2% every 2 min thereafter until volitional fatigue. Treadmill speed was selected based on the individual subject's training history and treadmill running experience to allow the test to last approximately 10–15 min and remained the same throughout the duration of the test. Metabolic and ventilatory variables were continuously monitored via open‐circuit, indirect calorimetry (Duke et al. 2014). Subjects breathed room air into a thermoplastic mouthpiece (Hans Rudolph, Kansas City, KS, USA) attached to a two‐way, non‐rebreathing valve. Two pneumotachographs (Hans Rudolph, Kansas City, KS, USA) were used to measure airflow on the inspired and expired sides, and the expired pneumotachograph was heated to allow for calculation of body, temperature, pressure, and saturation values despite changes in expired gas temperature during exercise. Two rapidly responding gas analyzers (Applied Electrochemistry, Pittsburgh, PA, USA) were used to analyze expired gases from a 5L mixing chamber for O_2_ and CO_2_ fractions. Data were recorded using offline data acquisition software (DasyLab, Measurement Computing, Norton, MA, USA) which allowed for calculation of $\dot{V}$O_2_, $\dot{V}$CO_2_, $\dot{V}$_E_, and RER.

### Determination of running speed at 70% of $\dot{V}$O_2max_

Running speed at 70% of $\dot{V}$O_2max_ was determined by having subjects run both with and without the weighted backpack on a flat (0% grade) treadmill at the same speed that was selected for the graded exercise test. Subjects ran for a minimum of 4 min at the prescribed speed until there was no change in $\dot{V}$O_2_ for two consecutive minutes. If necessary, treadmill speed was then adjusted if the observed $\dot{V}$O_2_ was either higher‐, or lower‐, than 70% of $\dot{V}$O_2max_. Following each change in speed, subjects ran a minimum of 4 min until there was no change in $\dot{V}$O_2_ for two consecutive min.

### Running time to exhaustion

During the running time to exhaustion trials (*visits* 2 and 3), subjects ran either with or without the weighted backpack on a 0% grade treadmill at the prescribed speed (eliciting 70% of $\dot{V}$O_2max_). Subjects were directed to run for the longest possible duration to the point of volitional exhaustion. All feedback about time and distance completed was withheld from subjects during the test. Exercise ventilatory and metabolic measures were recorded during the 8th min of exercise, and expiratory flow limitation was determined using methods full described previously (Duke et al. 2014). Briefly, the preexercise resting maximal flow‐volume loop was recorded as the composite of the best of three maximal forced vital capacity maneuvers and graded loops at 90%, 80%, 70%, 60%, and 50% effort. A composite maximal flow‐volume loop was utilized to account for thoracic gas compression and was generated by an in‐house data analysis program specifically designed for this purpose (Clipper 5.2, Computer Associates, Islandia, NY, USA). Exercise tidal flow‐volume relationships were collected during the final 30 sec of the 8th min of exercise. Inspiratory reserve volume (IRV) and expiratory reserve volume (ERV) were estimated by having subjects perform inspiratory capacity maneuvers at 30 and 55 sec of the 8th min of exercise. Forced vital capacity maneuvers were repeated immediately postexercise to account for exercise induced bronchodilation, and the best composite loop among the pre‐ and postexercise maximal flow‐volume loops was selected. Exercise tidal breath loops were placed in the proper position on the volume axis within the maximal flow‐volume loop using the same in‐house data analysis program described above. Expiratory flow limitation was calculated as a percentage of the expired tidal volume that met or exceeded the maximal flow‐volume loop (Duke et al. 2014). *T*_lim_ was recorded and diaphragm twitch pressure pre‐ and postexercise was recorded. We chose to measure ventilatory measures early in the exercise bout because minute ventilation has been shown to progressively increase during prolonged unloaded (Hopkins et al. 1998; Stickland et al. 2004) and loaded exercise (Phillips et al. 2016) and we therefore sought to minimize variability between subjects by comparing ventilatory measures at a standardized time early in the exercise bout. Therefore, we chose to compare ventilatory and metabolic measures recorded at the 8th minute of exercise, a time point at which subjects had been exercising long enough for ventilatory and metabolic measures to stabilize, but prior to the beginning of any upward drift consequent to prolonged exercise. However, we note that these measurements are not reflective of all stages of exercise.

### Load carriage instrumentation

Subjects exercised with a commercially available backpack (North Face Terra 50, Alameda, CA, USA). This type of backpack is commonly used by backpackers, day hikers, and other recreational enthusiasts. The appropriate size backpack was determined for each subject according to manufacturer directions and subjects were allowed to adjust the shoulder, sternum, hip, and stabilizing straps to mimic how they would wear the backpack for an extended hike. Strap placement was measured and recorded to the nearest millimeter to allow for duplication in subsequent trials. All testing was conducted with both the hip belt and sternum strap fastened to simulate a real‐life situation. Weights were placed in the backpack according to common practice, with lower mass items placed in the bottom of the pack and heavier items positioned in the middle of the pack closest to the subjects' back. In order to minimize shifting of the pack contents, other low‐mass items were inserted until the pack was filled to near its full capacity.

### Pulmonary and respiratory muscle function

Pulmonary and respiratory muscle function testing was conducted according to American Thoracic Society recommendations (Standardization of Spirometry, 1995) using a calibrated computerized spirometer (Vmax Encore, CareFusion, San Diego, CA), and two calibrated pneumotachographs (Hans Rudolph, Kansas City, KS, USA). Forced vital capacity (FVC), forced expiratory volume in 1 sec (FEV_1.0_), peak expiratory flow (PEF) were determined in triplicate from the maximal flow‐volume maneuvers as previously described (Duke et al. 2014). If either FVC or FEV_1.0_ values varied by more than 0.2 L, additional trials were completed until three reproducible trials were achieved. The highest values are reported. Maximal inspiratory mouth pressure (*P*_Imax_), and maximal expiratory mouth pressure (*P*_Emax_) were recorded pre‐ and post‐IMT. Maneuvers for *P*_Imax_ and *P*_Emax_ were initiated from residual lung volume and total lung capacity, respectively, and sustained for a minimum of 1 sec according to published guidelines (ATS/ERS Statement on Respiratory Muscle Testing, 2002). Subjects repeated the maneuvers until three trials within 10% of each other were observed. The highest values are reported.

### Perception of effort and dyspnea measures

Whole‐body ratings of perceived exertion (RPE, 6–20 scale) and dyspnea ratings (modified Borg scale, 0–10 scale) were obtained from subjects after 8 min of exercise and upon termination of exercise in both trials (Borg 1982). Dyspnea ratings on this scale are strongly correlated with the level of $\dot{V}$_E_ and have been demonstrated to be a highly reproducible measure of breathlessness (Wilson and Jones 1991). Subjects were shown separate visual analog scales for RPE and dyspnea, and instructed to provide their ratings on each scale during the 8th min of exercise, and at the point of volitional exhaustion in both running time to exhaustion trials.

### Diaphragm fatigue measurement

Esophageal and gastric pressures (*P*_es_ and *P*_ga_, respectively) were measured according to previously described methods (Milic‐Emili et al. 1964; Guenette et al. 2007; Dominelli et al. 2012) using balloon tipped latex catheters (no. 47‐9005; Ackrad Laboratory, Cranford, NJ) and a piezoelectric pressure transducer (Validyne Engineering, Northridge, CA, USA). A local anesthetic was applied to the nostril (lidocaine hydrochloride 2%, Xylocaine, AstraZeneca, Wilmington, Delaware), and two balloon‐tipped catheters were placed in the stomach, approximately 45 cm past the nostril. The second of the two catheters was withdrawn until a negative pressure was observed upon inspiration, then withdrawn a further 10 cm to ensure placement completely within the esophagus. Air was removed from both balloons using a glass syringe and by having subjects perform a Valsalva maneuver. Then 1 and 2 mL of air was injected into the esophageal and gastric balloons, respectively. The difference between *P*_es_ and *P*_ga_ was calculated and recorded as transdiaphragmatic pressure (*P*_di_).

Bilateral magnetic stimulation of the phrenic nerve was used to assess diaphragmatic fatigue (Similowski et al. 1989) using two 45‐mm figure‐eight coils attached to two magnetic stimulators (Magstim 200, MagStim, Whitland, UK) as previously described (Topeli et al. 1999). Briefly, the coils were placed around the posterior border of the sternomasoid muscles at the level of the cricoid cartilage and triggered simultaneously while subjects rested quietly at functional residual capacity (FRC). Stimulations began at 50% of maximal stimulator power output and progressively increased to 80% of max power by increments of 10%, followed by increases of 5% to 100% of max power. Plateaus in *P*_di, tw_ with increasing power output were taken as an indication that the phrenic nerves were maximally stimulated. Eight non‐potentiated twitches at 100% of stimulator power output separated by at least 30 sec were delivered while subjects were at FRC. Twitch amplitude (*P*_di, tw_) was monitored, and if the twitch amplitude continued to rise after the first two stimulations, the first two twitches were discarded. If *P*_es_ indicated that the subjects' FRC deviated from resting, individual twitches were also discarded. Diaphragmatic fatigue was considered present if a postexercise reduction greater than or equal to 10% in *P*_di, tw_ compared with baseline was observed (Verges et al. 2007).

### Flow‐resistive inspiratory muscle training protocol

Subjects were randomly assigned to either a true IMT group (IMT) or a placebo‐IMT group (PLA). Both groups performed training using a previously established test of incremental respiratory endurance (TIRE) regimen (Chatham et al. 1999; Gething et al. 2004; Mickleborough et al. 2010). The training protocol for both groups involved three training sessions per week for a period of 6 weeks using a flow‐resistive inspiratory muscle training device (PrO2Fit, Smithfield, RI, USA). The training device consisted of a hand‐held unit that interfaced with a tablet or smartphone via a wireless Bluetooth connection. Each training session began with three maximal inspiration maneuvers set against a resistance (a 2 mm leak in the device), with each inspiration initiated from residual lung volume. The associated software with the training device then calculated a training template that corresponded to either 80% of the sustained maximal inspiratory pressure (SMIP) during the best of the three maximal maneuvers for the TRT group, or 30% of SMIP for the PLA group, a workload which has been shown to have no training effect (Mickleborough et al. 2008; Shei et al. 2016b). Following completion of the baseline breaths, subjects were prompted to complete six forceful breaths, initiated from residual lung volume, to match the training template. The initial six training breaths were separated by 40 sec of rest. Following the sixth breath, the rest period was decreased to 30 s for the next six breaths, then to 20‐, 15‐, 10‐, and 5‐sec, for every six breaths. The training session was terminated if subjects were unable to match at least 90% of the training template for two successive breaths. The computer software recorded each training session and compliance was assessed by summing the number of recorded sessions for each subject and dividing by the total number of prescribed sessions.

### Data analysis

Statistical analysis was conducted using SPSS 24.0 (IBM Corporation, Chicago, IL, USA) statistical software. Data were assessed for normality using the Shapiro‐Wilk test and homogeneity of variances was confirmed with Levene's test. Mauchly's test was performed to assess sphericity of data. *T*_lim_, ventilatory and perceptual responses, and postexercise changes in *P*_di, tw_ at baseline were compared between the LC and UL conditions using paired *t*‐tests. To determine the effects of IMT, the LC condition (performed during either *visit 2 or visit 3*) was analyzed as the pretraining time point, and *visit 4* was analyzed as the posttraining time point. A split‐plot 2 × 2 (time [pre vs. post] by group [IMT vs. CON]) ANOVA was employed to determine the effect of the training intervention on the physiological variables measured. Significant main effects were further explored using Bonferroni's post hoc test for multiple comparisons. A non‐parametric Related‐Samples Wilcoxon Signed‐Rank Test was used to detect differences in *T*_lim_ between pre‐ and post‐IMT data because the data were not normally distributed. Pearson correlations were determined for diaphragmatic fatigue after the six‐week training period (%Δ in *P*_di, tw_ from pre‐ to postexercise in the final trial) on %Δ in *T*_lim_ from pre‐ to posttraining as well as for %Δ in *P*_Imax_ from pre‐ to posttraining on %Δ in *T*_lim_ from pre‐ to posttraining. Significance was accepted at *P *<* *0.05. All data are given as mean(SD) with the exception of the difference in *T*_lim_ from pre‐ to posttraining, which is given as median and range because a non‐parametric test was used. Sample size was based on an *a priori* power analysis (G*Power 3.1.3, Franz Faul, Germany) of each dependent measure (Faghy and Brown 2016), at a power of 0.8 (*d *=* *0.86).

## Results

### Subjects

No significant differences were observed between groups in height, body mass, age, body mass index, $\dot{V}$O_2max_, weekly activity, or adherence to the prescribed IMT training (*P *>* *0.05; Table 1).

### Exercise tolerance

**Figure 1:**
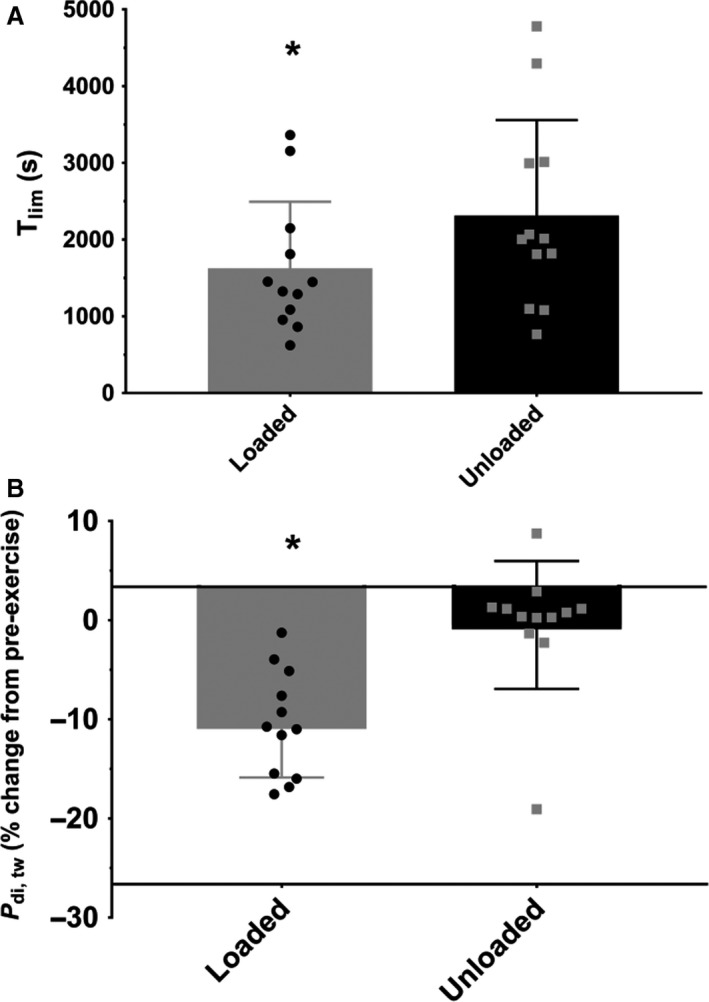
*T*_lim_ (Panel A) and *P*_di, tw_ (Panel B) data for LC and UL conditions at baseline. Data are given as mean(SD). *significantly different between LC and UL. *T*_lim_ was 42.9(29.1)% shorter (*P *<* *0.001, 95% CI: −990.4 to −379.9 sec) with thoracic LC (1626.5 (866.7) sec) compared to UL (2311.6 (1246.5) sec). The postexercise reduction in *P*_di, tw_ was significantly greater in LC (−13.9 (5.3)%), but not in UL (−3.8 (6.5)%); *P *=* *0.001, %Δ, +261.3; 95% CI: −14.7 to −5.3%.

**Figure 2:**
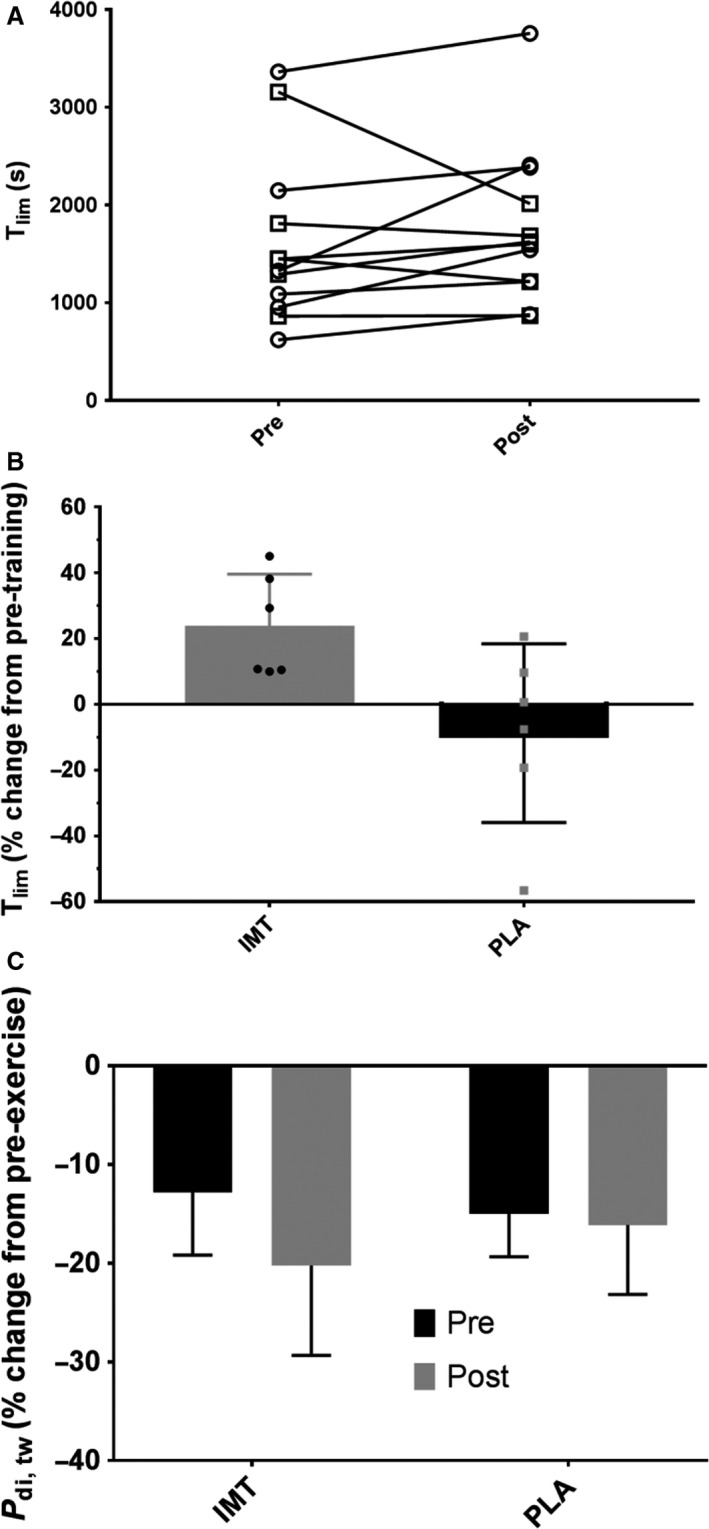
Pre‐ and Posttraining *T*_lim_ for each group (open circles, IMT, open squares PLA). *T*_lim_ (Panel B) and *P*_di, tw_ (Panel C) data from pre‐ to postIMT. *significantly different from pre‐ to posttraining. *T*_lim_ in the IMT group improved significantly more (median change 20.0%, range +9.9 to +45%; *P *=* *0.046, 95% CI +3.7 to +65.1%) compared to the PLA group (median change −3.5%, range −56.6 to +20.6%).

Subjects ran significantly slower (Table 2) and *T*_lim_ was significantly reduced in the thoracic LC condition compared with the UL condition (Fig. 1). At baseline *T*_lim_ did not differ between training groups (*P *=* *0.600, 95% CI: −1282.8 to +1205.0 sec; IMT group 1583.2(1012.1) sec, PLA group 1669.79(789.9) sec). Post‐IMT, *T*_lim_ was significantly improved in the IMT group compared to PLA (Fig. 2). The %Δ in *P*_Imax_ from pre‐ to posttraining was significantly correlated with the %Δ in *T*_lim_ from pre‐ to posttraining (*P *=* *0.031, *r *=* *0.62). Absolute $\dot{V}$O_2_ did not differ between LC and UL (Table 2), between IMT and PLA at baseline, and between IMT and PLA posttraining (Table 3, all *P *>* *0.05).

**Table 2:** Running speed, ventilatory, and perceptual data at baseline

Variable	LC	UL	*P*‐value	%Δ	95% CI
Running Speed (km·hr^−1^)	9.5 (1.1)^a^	11.4 (1.4)	<0.001	16.6	−2.3 to −1.5
At 8th minute of exercise					
$\dot{V}$O_2_ (L·min^−1^)	3.12 (0.38)	3.12 (0.37)	0.953	–	−0.06 to +0.06
$\dot{V}$_E_ (L·min^−1^)	88.3 (14.2)^a^	82.9 (12.7)	0.004	6.1	+2.1 to +8.7
*V*_T_ (L·breath^−1^)	2.01 (0.4)	2.20 (0.4)	0.112	–	−0.43 to +0.05
*f*_b_ (breaths·min^−1^)	45 (5.5)^a^	39 (7.9)	0.029	13.0	+0.7 to +10.8
EFL (%)	26.4 (31.3)	18.2 (23.2)	0.239	–	−6.3 to +22.8
IRV (L)	1.4 (0.6)	1.5 (0.5)	0.727	–	−0.3 to +0.2
(% FVC)	27.2 (9.0)	28.1 (8.7)	–	–	–
ERV (L)	1.0 (0.5)^a^	1.3 (0.7)	0.033	−25.9	−0.6 to −0.03
(% FVC)	18.0 (8.3)	24.4 (12.1)	–	–	–
T_i_/T_Total_ (%)	51.4 (2.9)	49.8 (3.7)	0.084	–	−0.3 to +3.5
RPE	13.2 (2.4)^a^	11.6 (2.5)	0.002	12.0	+0.7 to +2.5
Dyspnea	4.3 (1.6)^a^	3.4 (1.6)	0.014	21.2	+0.2 to +1.6
At end‐exercise (*T*_lim_)					
RPE	15.8 (2.1)	15.3 (2.3)	0.096	–	−0.1 to +0.9
Dyspnea	5.9 (1.6)	5.5 (1.8)	0.096	–	−0.1 to +0.9

$\dot{V}$O_2_, absolute oxygen consumption; $\dot{V}$_E_, minute ventilation; *V*_T_, tidal volume; *f*_b_, breathing frequency; EFL, expiratory flow limitation; IRV, inspiratory reserve volume; ERV, expiratory reserve volume; T_i_/T_Total_, inspiratory duty cycle; RPE, ratings of perceived exertion.

a Significantly different from UL, *P* < 0.05. Data are given as mean(SD).

**Table 3:** Metabolic, ventilatory, and perceptual data

Variable	IMT		PLA	
	Pretraining	Posttraining	Pretraining	Posttraining
At 8th minute of exercise				
$\dot{V}$O_2_ (L·min^−1^)	3.15 (0.40)	3.13 (0.42)	3.08 (0.40)	3.10 (0.41)
$\dot{V}$_E_ (L·min^−1^)	91.0 (19.2)	88.50 (18.4)	85.7 (7.9)	82.7 (8.6)
*V*_T_ (L·breath^−1^)	2.12 (0.53)	1.91 (0.33)	1.90 (0.22)	1.81 (0.30)
*f*_b_ (breaths·min^−1^)	44 (6.5)	46 (6.6)	45 (4.8)	46 (4.5)
EFL (%)	24.0 (18.2)	21.0 (26.1)	28.9 (42.6)	29.9 (39.5)
IRV (L)	1.6 (0.7)	1.4 (0.6)	1.3 (0.4)	1.5 (0.3)
(% FVC)	28.5 (10.5)	26.2 (10.0)	26.0 (7.6)	30.2 (6.4)
ERV (L)	1.2 (0.4)	1.2 (0.3)	0.8 (0.5)	0.7 (0.4)
(% FVC)	21.2 (6.4)	22.4 (5.6)	14.8 (9.1)	13.3 (6.1)
T_i_/T_Total_ (%)	52.0 (3.4)	49.7 (2.7)	50.7 (3.4)	49.2 (2.1)
RPE	12.2 (2.9)	12.8 (2.9)	14.2 (1.5)	12.8 (1.6)
Dyspnea	4.0 (1.9)	3.8 (1.2)	4.7 (1.2)	4.2 (1.2)
At end‐exercise (*T*_lim_)				
RPE	15.3 (2.5)	15.8 (2.4)	16.2 (1.7)	15.5 (2.5)
Dyspnea	5.8 (1.7)	5.2 (1.6)	6.0 (1.6)	5.8 (2.1)

IMT, inspiratory muscle training group; PLA, placebo group; $\dot{V}$O_2_, absolute oxygen consumption; $\dot{V}$_E_, minute ventilation; *V*_T_, tidal volume; *f*_b_, breathing frequency; EFL, expiratory flow limitation; IRV, inspiratory reserve volume; ERV, expiratory reserve volume; T_i_/T_Total_, inspiratory duty cycle; RPE, ratings of perceived exertion. Data are given as mean(SD).

### Ventilatory measures

Ventilatory data are presented in Tables 2 and 3. Data from the 8th min of exercise are presented to demonstrate any differences in $\dot{V}$_E_ that may have occurred during fixed‐workload exercise when $\dot{V}$O_2_ was matched between conditions and had plateaued during the early stages of the exercise bout. $\dot{V}$_E_ and *f*_b_ during the 8th min of exercise were significantly higher in the thoracic LC condition compared to the UL condition, but *V*_T_ was unchanged. Expiratory flow limitation, inspiratory duty cycle (T_i_/T_Total_), and IRV were not significantly different between conditions. ERV was significantly lower in the thoracic LC trial compared to the UL trial. No significant main effects were observed in $\dot{V}$_E,_ *f*_b_, or *V*_T_ during the 8th min of exercise (*P *>* *0.05) from pre‐ to post‐IMT. No significant main effects were observed in expiratory flow limitation, T_i_/T_Total_, IRV, or ERV (*P *>* *0.05) from pre‐ to post‐IMT.

### Diaphragmatic fatigue

The change in *P*_di, tw_ from pre‐ to postexercise was significantly greater in the thoracic LC condition compared with the UL condition (Fig. 1). In the thoracic LC condition, nine subjects had postexercise reductions in *P*_di, tw_ ≥ 10% (i.e., diaphragmatic fatigue present) while three subjects had postexercise reductions in *P*_di, tw_ <10%. In the UL condition, one subject had a postexercise reduction in *P*_di, tw_ ≥10%, and 11 subjects had a postexercise reduction in *P*_di, tw_ <10%.

The change in *P*_di, tw_ from pre‐ to postexercise was similar in both the pre‐ and post‐IMT trials (Fig. 2). No significant differences were observed between groups at baseline and posttraining (*P *>* *0.05). Furthermore, no differences were observed from pre‐ to posttraining within either group (*P *>* *0.05). *P*_di, tw_ was not significantly correlated with the %Δ in *T*_lim_ from pre‐ to posttraining (*P *=* *0.507, *r *=* *−0.213), or with the %Δ in *P*_Imax_ from pre‐ to posttraining (*P *=* *0.819, *r *=* *−0.074).

### Perception of effort and dyspnea

RPE and dyspnea at the 8th min of exercise were significantly greater in the LC condition compared to the UL condition (Table 2). However, RPE and dyspnea at *T*_lim_ did not differ between the LC and UL conditions.

No significant main effects from pre‐ to post‐IMT were observed for RPE at the end of exercise (Table 3). At the 8th min of exercise, no significant main effects from pre‐ to post‐IMT for treatment or time were observed for RPE (*P *>* *0.05), however a significant treatment by time interaction was observed in RPE at the 8th min of exercise (*P *=* *0.033). Pairwise comparisons revealed that RPE at the 8th min of exercise was significantly lower posttraining than pretraining in the PLA group (*P *=* *0.025). No other pairwise comparisons showed any significant differences (*P *>* *0.05). No significant main effects were observed in dyspnea ratings at the 8th min of exercise or at the end of exercise (*P *>* *0.05).

### Pulmonary function

No significant differences were observed in FVC, FEV_1.0_, or PEF between groups at baseline (*P *>* *0.05, Table 4). Following the six‐week training period, no significant differences were observed in FVC, FEV_1.0_, or PEF between groups, or from baseline in either group (*P *>* *0.05, Table 4).

**Table 4:** Pulmonary function data

Variable	IMT		PLA	
	Pretraining	Posttraining	Pretraining	Posttraining
FVC (L)	5.5 (0.5)	5.2 (0.6)	5.1 (0.6)	5.1 (0.7)
FEV_1.0_ (L)	4.0 (0.4)	4.0 (0.4)	3.9 (1.0)	3.8 (0.5)
PEF (L·s^−1^)	10.0 (0.6)	9.9 (0.6)	9.5 (2.3)	9.7 (1.6)
*P*_Imax_ (cmH_2_O)	145.5 (24.5)	163.3 (24.2)^a^	135.3 (41.5)	128.7 (38.6)
*P*_Emax_ (cmH_2_O)	166.3 (51.4)	187.5 (45.3)^a^	114.0 (48.3)	116.5 (56.4)

IMT, inspiratory muscle training training group; PLA, placebo training group; FVC, forced vital capacity; FEV_1.0_, forced expiratory volume in 1 sec; PEF, peak expiratory flow; *P*_Imax_, maximal inspiratory mouth pressure; *P*_Emax_, maximal expiratory mouth pressure. Data are given as mean(SD).

a Significantly different from pretraining; IMT group *P*_Imax_ difference pre‐ to posttraining *P *=* *0.003, 95% CI +9.0 to +26.6 cmH_2_O; IMT group *P*_Emax_ difference pre‐ to posttraining *P *=* *0.012, 95% CI +7.1 to +35.3 cmH_2_O.

### Respiratory Muscle Strength

*P*_Imax_ and *P*_Emax_ did not differ between groups at baseline (*P *>* *0.05, Table 4). Following the 6 week training period, *P*_Imax_ and *P*_Emax_ were significantly increased in the IMT group (*P *<* *0.05), but not the PLA group (*P *>* *0.05, Table 4).

## Discussion

We have demonstrated for the first time that (1) diaphragmatic fatigue occurs following constant‐load running to volitional exhaustion with thoracic LC, as indicated by a significant postexercise reduction in *P*_di, tw_, and (2) six weeks of flow‐resistive IMT improved running time to exhaustion with thoracic LC. While inspiratory muscle strength (i.e., *P*_Imax_) increased following 6 weeks of flow‐resistive IMT, diaphragmatic fatigue and ventilatory mechanics following a constant workload running test to exhaustion with thoracic LC, were unchanged relative to preflow resistive IMT (baseline) values. Our data suggest that flow‐resistive IMT may have delayed the development of diaphragmatic fatigue (>10% decline in *P*_di, tw_ postexercise), thereby allowing subjects to run for a longer duration.

Our observations of reduced exercise capacity, diaphragmatic fatigue, elevated $\dot{V}$_E_, and altered breathing mechanics with thoracic LC highlight the specific stress that LC places on the pulmonary system. These findings, which are consistent with published data (Faghy and Brown 2014, 2016; Faghy et al. 2016; Phillips et al. 2016), may partially account for the deleterious effects of thoracic LC on exercise capacity. The magnitude of diaphragmatic fatigue we observed (%Δ −13.9%), measured using the most advanced technique available (diaphragmatic twitch), confirms the finding of global respiratory muscle fatigue by Faghy and Brown (2014) using voluntary mouth pressure measures (%Δ ‐11%). The increase in diaphragmatic fatigue and $\dot{V}$_E_ with thoracic LC are likely a consequence of an increase in the work of breathing and impairment of breathing mechanics imposed by thoracic LC (Tomczak et al. 2011; Brown and McConnell 2012; Dominelli et al. 2012). We observed an elevated $\dot{V}$_E_ and lower ERV, which is in agreement with previous findings (Dominelli et al. 2012; Phillips et al. 2016), but no difference in the severity of expiratory flow limitation. The elevated $\dot{V}$_E_ may have been a consequence of increased dead space ventilation, which has been demonstrated previously (Phillips et al. 2016), and was accompanied by an increase in *f*_b_. This finding suggests that the subjects may have adopted a rapid and shallow breathing pattern to minimize the elastic work of breathing (Phillips et al. 2016). Furthermore, given that expiratory flow limitation did not differ between loaded and unloaded conditions, ventilatory demand at the workloads in both baseline trials was likely low enough that subjects had a substantial ventilatory reserve. If so, subjects were likely able to breathe at an operational lung volume that minimized expiratory flow limitation (Babb 2013).

Importantly, we note that this is the first study to document diaphragmatic fatigue following exercise to volitional exhaustion at 70% of $\dot{V}$O_2max_ with thoracic LC, but not following unloaded exercise at an equivalent absolute $\dot{V}$O_2_. Historically, diaphragmatic fatigue is well documented only in unloaded exercise to exhaustion at or above 85% of $\dot{V}$O_2max_ (Harms et al. 1997; Romer et al. 2006) or prolonged duration exercise lasting more than 208 ± 22 min (Ross et al. 2008). However, given that we observed diaphragmatic fatigue following exercise to exhaustion at 70% of $\dot{V}$O_2max_ with a mean duration of less than 30 min, we propose that thoracic LC lowers the critical threshold at which diaphragmatic fatigue occurs compared with unloaded exercise.

Our data shows that 6 weeks of flow‐resistive IMT improved exercise capacity in a submaximal constant‐load treadmill running task with thoracic LC. The observed improvement confirms that those seeking to improve their ability to sustain submaximal workloads for prolonged periods of time will benefit from flow‐resistive IMT. While others have demonstrated an ergogenic effect of 6 weeks of pressure‐threshold IMT on running time trial performance with LC (Faghy and Brown 2016) and improved unloaded exercise capacity in recreational runners (Mickleborough et al. 2010), our novel finding suggests that IMT can be applied as an ergogenic aid for recreational and occupational personnel whose activities require sustained exercise with thoracic LC. The magnitude of exercise capacity improvement in our current study was greater that the ~8% improvement observed by Faghy and Brown (2016) and 16.4% improvement in our previous study (Mickleborough et al. 2010), which may be a consequence of the chosen criterion task. In our current study, subjects ran to volitional exhaustion with LC at 70% of $\dot{V}$O_2max_, whereas in our previous study subjects performed the run to exhaustion in an unloaded condition at 80% of $\dot{V}$O_2max_ and Faghy and Brown (2016) employed a 2.4 km running time trial. These differences in exercise tasks may, in part, account for the varied magnitudes of the ergogenic effects of IMT on exercise performance.

The mechanisms behind the improved exercise capacity with IMT are likely related to increases in inspiratory muscle strength and endurance (McConnell 2012). Subjects in the IMT group had significantly greater inspiratory muscle strength post‐IMT as assessed by *P*_Imax_, whereas *P*_Imax_ was unchanged in the PLA group. Furthermore, the improvement in *T*_lim_ was significantly correlated with increases in *P*_Imax_. IMT has been documented to promote hypertrophy in the diaphragm and external intercostals as well as increase the proportion of type II muscle fibers in the external intercostals (Shei et al. 2016a). The adaptations to IMT have been shown to decrease the perception of dyspnea, reduce blood lactate concentration (Mickleborough et al. 2010), increase efficiency of the respiratory muscle pump (Turner et al. 2012), and attenuate the respiratory muscle metaboreflex (Witt et al. 2007), which is a sympathetically mediated vasoconstriction that shunts blood away from the limb locomotor muscles that has been shown to impair exercise performance (Harms et al. 1997; Dempsey et al. 2006). Taken together, these adaptations may partially account for the observed ergogenic effects of IMT on exercise capacity with thoracic LC. However, we note that no direct measures of these factors other than the perception of dyspnea, which was unchanged, were recorded, thus the mechanistic basis of the improvement in performance in this study remains speculative.

In contrast to our hypothesis, postexercise diaphragmatic fatigue was unchanged following IMT. The exercise task in this study required subjects to exercise to volitional exhaustion. Exercising to the point of volitional exhaustion likely required subjects to exercise past the point at which diaphragmatic fatigue develops, which may partially explain the lack of change in postexercise diaphragmatic fatigue. It is plausible that with constant‐load exercise to volitional exhaustion, rather than reduce the magnitude of diaphragmatic fatigue, IMT delays the rate of development of diaphragmatic fatigue by improving inspiratory muscle strength and endurance (McConnell 2012). We observed that changes in *P*_Imax_ from pre‐ to posttraining were correlated with changes in *T*_lim_ from pre‐to posttraining, suggesting an association between improvements in inspiratory muscle strength and exercise capacity with thoracic LC.

### Methodological considerations

In order to protect our subjects from injury in the event of an accidental fall while running, the esophageal balloon‐tipped catheters were left in place (i.e., subjects remained intubated), but disconnected from the pressure‐transducers and secured to a headgear piece worn by subjects during the running time to exhaustion trials. Therefore, we did not measure the work of breathing during the running time to exhaustion trials and cannot definitively attribute the increased diaphragmatic fatigue with thoracic LC to an increase in the work of breathing. Despite this limitation, we believe it is reasonable to speculate based on previous studies (Tomczak et al. 2011; Dominelli et al. 2012) that even at the same relative intensity, the work of breathing is likely greater with thoracic LC compared with UL due to changes in respiratory mechanics and the elastic resistance imposed by the thoracic LC. Therefore, further studies are required to describe the effect of thoracic LC on the work of breathing during exercise at the same relative intensity.

A second limitation of this study was that no indices of the respiratory muscle metaboreflex were recorded. While it is possible that the diaphragmatic fatigue in the thoracic LC condition could have triggered a respiratory muscle metaboreflex (Dempsey et al. 2006), we note that (1) the impairment in exercise capacity with thoracic LC cannot be definitively attributed to it, and (2) the role of the respiratory muscle metaboreflex in improving exercise capacity following 6 weeks of flow‐resistive IMT is yet unclear. Thus, the relationship between the respiratory muscle metaboreflex, thoracic LC, and the influence of IMT on these factors presents a fertile area for future study.

## Conclusions

In summary, our novel findings demonstrate that 6 weeks of flow‐resistive IMT improves exercise capacity with thoracic LC. The improvement in exercise performance could be a consequence of increased inspiratory muscle strength, which may be associated with delays in the onset of diaphragmatic fatigue. We have also shown that thoracic LC (10 kg) during constant‐load sub‐maximal running exercise to exhaustion induced diaphragmatic fatigue and impaired exercise capacity compared to unloaded exercise. These findings indicate that personnel seeking to improve exercise capacity with thoracic LC may adopt IMT as an ergogenic training intervention. Both occupational and recreational situations that require LC may benefit from incorporating IMT into their normal exercise routine.

## Conflict of Interest

The authors declare no conflicts of interest.
